# The Genetics and Biochemistry of Cell Wall Structure and Synthesis in *Neurospora crassa*, a Model Filamentous Fungus

**DOI:** 10.3389/fmicb.2019.02294

**Published:** 2019-10-10

**Authors:** Pavan K. Patel, Stephen J. Free

**Affiliations:** Department of Biological Sciences, SUNY University at Buffalo, Buffalo, NY, United States

**Keywords:** cell wall, filamentous fungi, Neurospora, glucan, galactomannan, mannanase, glucanosyltransferase, melanin

## Abstract

This review discusses the wealth of information available for the *N. crassa* cell wall. The basic organization and structure of the cell wall is presented and how the wall changes during the *N. crassa* life cycle is discussed. Over forty cell wall glycoproteins have been identified by proteomic analyses. Genetic and biochemical studies have identified many of the key enzymes needed for cell wall biogenesis, and the roles these enzymes play in cell wall biogenesis are discussed. The review includes a discussion of how the major cell wall components (chitin, β-1,3-glucan, mixed β-1,3-/ β-1,4- glucans, glycoproteins, and melanin) are synthesized and incorporated into the cell wall. We present a four-step model for how cell wall glycoproteins are covalently incorporated into the cell wall. In *N. crassa*, the covalent incorporation of cell wall glycoproteins into the wall occurs through a glycosidic linkage between lichenin (a mixed β-1,3-/β-1,4- glucan) and a “processed” galactomannan that has been attached to the glycoprotein N-linked oligosaccharides. The first step is the addition of the galactomannan to the N-linked oligosaccharide. Mutants affected in galactomannan formation are unable to incorporate glycoproteins into their cell walls. The second step is carried out by the enzymes from the GH76 family of α-1,6-mannanases, which cleave the galactomannan to generate a processed galactomannan. The model suggests that the third and fourth steps are carried out by members of the GH72 family of glucanosyltransferases. In the third step the glucanosyltransferases cleave lichenin and generate enzyme/substrate intermediates in which the lichenin is covalently attached to the active site of the glucanosyltransferases. In the final step, the glucanosyltransferases attach the lichenin onto the processed galactomannans, which creates new glycosidic bonds and effectively incorporates the glycoproteins into the cross-linked cell wall glucan/chitin matrix.

## Introduction

The cell wall is a vital structure for virtually all fungal cells. The wall provides protection from environmental stresses such as UV light, desiccation, freezing, and attack from enzymes that might otherwise cause cell lysis. It provides the tensile strength required to protect the cell against cell lysis from osmotic pressure. It facilitates adhesion to the substratum. Receptors in the cell wall allow the fungus to assess a large variety of environmental conditions and to activate cell signaling pathways. The cell wall is also the major determinant of fungal cell morphology. Mutations affecting cell wall synthesis affect the growth rate, morphology, and viability of fungal cells.

The major cell wall components include glucans, glycoproteins, and chitin (Klis et al., 2006; Latge, 2007; Chaffin, 2008; Free, 2013; Gow et al., 2017). Almost all fungal cell walls contain β-1,3-glucan (laminarin), chitin, and a variety of glycoproteins that function in cell wall biogenesis, adhesion, environmental sensing, and as cell wall structural elements. In addition to these general components, fungal cell walls often contain additional polysaccharides such as α-1,3-glucan, β-1,6-glucan, mixed β-1,3-/β-1,4-glucans, galactomannans, xylogalactomannans, and other less well-characterized glucans. Many fungi incorporate melanin into their cell walls. While we will address each of these various components individually, it is important to recognize that they are all cross-linked together and function as an assemblage.

Fungal cell walls are dynamic structures. Their composition is responsive to environmental changes. The well-characterized cell wall integrity signal transduction pathway is a signaling pathway for modifying the cell wall under stress conditions. When activated, the cell wall integrity pathway directs the synthesis of addition cell wall glycoproteins and an increase in cell wall chitin and glucans. The filamentous fungi have life cycles that include a variety of different cell types. It is clear that the cell wall can be dramatically changed as different types of cell are generated during fungal life cycles and cell type-specific cell wall proteins and glucans are expressed.

While a great deal of information is available on the cell walls from a number of fungi, this review is focused on the cell walls from the model filamentous fungus *Neurospora crassa*. Pertinent information is available about *N. crassa* cell walls from vegetative hyphae, from conidia (asexual spores), from cells in the perithecium (female mating structure), and from the developing ascospores (sexual spores) (Bowman et al., 2006; Maddi et al., 2009; Ao et al., 2016). The fungus therefore presents a broad overview of cell wall structures and serves as an excellent model for the characterization of cell wall structure and biosynthesis. Neurospora is particularly well suited for the study of the fungal cell wall. *N. crassa* is a haploid fungus, which greatly facilitates the isolation and characterization of mutants affected in the generation of the cell wall. *N. crassa* is currently the only filamentous fungus with a nearly complete single gene knockout library, and mutants lacking almost any gene of interest are readily available from the Fungal Genetics Stock Center (Colot et al., 2006). The knockout library has proven to be a valuable resource for the characterization of *N. crassa* cell walls. The library allows an investigator to rapidly determine if a putative cell wall protein or a polysaccharide synthase plays an important role in generating the cell wall for all of the different cell types in the *N. crassa* life cycle. The tools for the genetic manipulation of *N. crassa* are well developed and have been immensely valuable in the characterization of cell wall glycoproteins. With all these advantages, *N. crassa* cell walls are among the best-characterized cell walls among the filamentous fungi. While this review concentrates on the genetics and biochemistry of *N. crassa* cell walls, some comparisons and contrasts with the cell walls of other fungi are included to illustrate elements that are in common among all cell walls and to point out features that may be unique to *N. crassa* and closely related fungal species.

In addition to the genetics and biochemistry of *N. crassa* cell wall biogenesis described in this article, a great deal is known about how chitin synthase, glucan synthase, and cell wall enzymes are being targeted to the hyphal tip, the locale where the cell wall is produced. The polysaccharide synthases and cell wall glycoproteins are trafficked through the Spitzenkorper, a densely packed region of intracellular vesicles that acts as a vesicle supply center to provide secretory vesicle to the hyphal tip. The Spitzenkorper has been shown to contain an inner area of chitin synthase-containing small microvesicles (chitosomes) at its core and a ring of larger macrovesicles surrounding the chitosome core. These macrovesicles have been shown to contain glucan synthase and cell wall enzymes. Both microvesicles and macrovesicles are targeted for fusion at the hyphal tip where cell wall formation occurs. An excellent review article detailing these aspects of *N. crassa* cell wall biogenesis has recently been published (Verdin et al., 2019). The reader is referred to that review article for more detailed information on vesicle trafficking of polysaccharide synthases to the plasma membrane and secretion of cell wall glycoproteins to the cell wall space.

## The Structures, Synthesis and Functions of *N. crassa* Cell Wall Components

**FIGURE 1:**
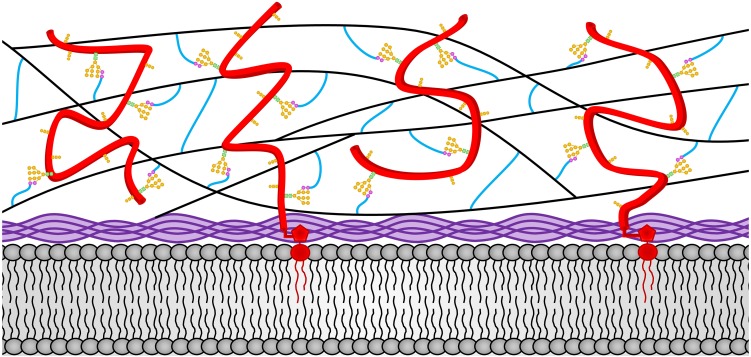
The *N. crassa* vegetative hyphae cell wall. The locations of the various cell wall components and how they are cross-linked together in the vegetative cell wall are depicted. Chitin is shown in purple and is located adjacent to the plasma membrane at the bottom of the diagram. The β-1,3-glucan is shown in black and located in the middle of the cell wall. Cell wall glycoproteins are shown in red. GPI anchors are shown in red and extent into the plasma membrane. N-linked oligosaccharides are shown with *N*-acetylglucosamine residues in green squares, mannoses from the N-linked oligosaccharide shown in orange circles, and processed galactomannans shown in magenta circles. O-linked oligosaccharides are also shown in orange. Lichenin is shown in blue and is attached to the processed galactomannan and to β-1,3-glucans. Note that the β-1,3-glucan, lichenin, and glycoproteins form a cross-linked cell wall matrix.

The *N. crassa* cell wall has been shown the contain β-1,3-glucan, mixed β-1,3-/β-1,4- glucans, α-1,3-glucan, chitin, melanin, and over forty different glycoproteins. We will discuss the structure and location of these *N. crassa* cell wall components within the cell wall structure. We also discuss how these components are made and incorporated into the cell wall. A representation of the *N. crassa* vegetative hyphal cell wall is shown in Figure 1.

## Chitin

Chitin is an important cell wall polysaccharide and is found in almost all fungal cell walls with the exception of the cell walls from *Schizosaccharomyces pombe* and *Pneumocystis* species (Magnelli et al., 2005; de Groot et al., 2007; Klis et al., 2010; Lenardon et al., 2010; Ma et al., 2016). Chitin is a long polysaccharide of repeating β-1,4-*N*-acetylglucosamine residues. Chitin makes up approximately 4% of the vegetative *N. crassa* cell wall mass (Aranda-Martinez et al., 2016). It is thought to be the major polysaccharide found in the *N. crassa* septae (Hunsley and Gooday, 1974). Multiple chitin polymers form interchain hydrogen bonds with each other and self-assemble into microfibrils, in which the individual chitin molecules are arranged in an antiparallel manner to create “crystalline” chitin (Ruiz-Herrera et al., 2006). The chitin is vital for the strength and integrity of the cell walls. It is localized in the membrane proximal portion of the cell wall and is incorporated into the wall matrix by being cross-linked to the glucans (Figure 1).

Fungi generally contain several genes encoding chitin synthases and vegetative hyphae express multiple chitin synthase genes. In the filamentous fungi, these chitin synthase genes are organized into seven different groups or classes, with the fungi having one or more genes from each of these seven classes (Choquer et al., 2004). It is thought that these chitin synthases may be producing chitin polymers of different lengths, at different cellular locations, and at different points in time across the fungal life cycles. Some chitin synthases have been shown to deposit chitin in the cell wall at the growing hyphal tip while other function to deposit chitin into the growing septum cell wall during septum formation (Roncero, 2002; Lee et al., 2004; Fukuda et al., 2009; Fajardo-Somera et al., 2015). All of the chitin synthases are thought to extrude chitin monomers into the cell wall space through a pore formed by their multiple transmembrane domains, and the formation of chitin fibrils occurs *in situ* in the cell wall space.

The *N. crassa* genome contains 7 chitin synthase genes, *chs-1/ncu03611*, *chs-2/ncu05239*, *chs-3/ncu04251*, *chs-4/ncu09324*, *chs-5/ncu04352*, *chs-6/ncu05268*, and *chs-7/ncu04350*, one from each of the seven groups commonly found in filamentous fungi (Fajardo-Somera et al., 2015). CHS-1, CHS-3, and CHS-4 were identified as being involved in cell wall synthesis by more classical genetic studies (Yarden and Yanofsky, 1991; Din and Yarden, 1994; Din et al., 1996). CHS-1, a class III chitin synthase, was found to be required for cell wall formation (Yarden and Yanofsky, 1991) and CHS-3, a class I chitin synthase, played an important role during vegetative growth. More recently, the cellular locations for all of these chitin synthases have been characterized and deletion mutants lacking each of these chitin synthases have been analyzed (Sanchez-Leon et al., 2011; Fajardo-Somera et al., 2015). Two of these chitin synthases, CHS-5 and CHS-7, contain a myosin-motor domain (MMD) and play important roles in apical growth, conidia development, and perithecia formation. All of the chitin synthases were shown to be localized in small vesicles, termed chitosomes, found in the Spitzenkorper, a region of vesicles just behind the growing hyphal tip that supplies vesicle for fusion at the hyphal tip. Slight differences in their locations suggests that they be located in different subpopulations of chitosomes. The chitin synthases were also found in association with developing septa, where cell wall is also being deposited. The septa are particularly well stained with Calcoflour white, a chitin staining reagent. The chitin synthases are also localized at the cross-walls between developing conidia, indicating a role in asexual development (Fajardo-Somera et al., 2015). Analysis of the chitin synthase deletion mutants showed that some of the chitin synthases were required for sexual development and loss of some of the chitin synthases affected vegetative growth and/or the production of conidia.

Many fungi contain chitin deacetylase enzymes which deacetylate chitin to form chitosan, which is much more soluble than chitin. Chitin deacetylases have been shown to generate chitosan during sporulation in *S. cerevisiae* (Christodoulidou et al., 1996). Chitosan formation has also been shown to be necessary for pathogenicity in *C. neoformans* (Baker et al., 2011). *N. crassa* has two chitin deacetylase genes, *ncu09508* and *ncu09582*, which may be more highly expressed during perithecium development (Lehr et al., 2014; Wang et al., 2014; Liu et al., 2017). No information is available about whether the perithecium contains chitosan or how the loss of the two chitin deacetylase genes affects female development.

## β-1,3-Glucan

β-1,3-glucan is a long unbranched polysaccharide consisting of repeating β-1,3-glucose residues. It is the most abundant component of the vegetative cell wall, making up approximately 35% of the *N. crassa* cell wall mass (Kar et al., 2019). It is the major component of the cell walls found in almost all fungi. β-1,3-glucan is well suited for its role as a major component of the fungal cell wall. The polymer has been shown to have a helical three-dimensional structure that allows for some limited stretching while retaining its structural integrity and its tensile strength (Bohn and Bemiller, 1995). The β-1,3-glucans are cross-linked together to form the basic three dimensional matrix structure of the wall. As such, β-1,3-glucans are found throughout the middle portion of the cell wall (Figure 1). The three dimensional β-1,3-glucan matrix would allow for a limited amount of cell wall stretching in all dimensions in response to the cell wall turgor pressure from within the cell. Direct measurements of the turgor pressure in *N. crassa* hyphae give values in the range of 500 pKa (70 psi) indicating that the hyphal cell wall is exposed to a significant amount of pressure (Lew, 2011). Sugar linkage analyses of the monosaccharides released from the *N. crassa* cell wall has failed to identify significant amounts of glucose with 1,6 linkages, while glucoses with 1,3 and 1,4 linkages are abundant (Maddi et al., 2009, 2012; Maddi and Free, 2010; Ao et al., 2016). This indicates that *N. crassa* does not make a β-1,6- polymer. The situation in *N. crassa* clearly differs from that found in the *S. cerevisiae* and *C. albicans* cell walls, where the β-1,6-glucan are used to cross-link the β-1,3-glucans together (Lu et al., 1995; Kollar et al., 1997; Kapteyn et al., 2000).

The FKS-1 β-1,3-glucan synthase is responsible for the synthesis of β-1,3-glucan, and the enzyme has been identified as being critical for cell wall formation in a number of fungi (Beauvais et al., 2001; Dichtl et al., 2015). An *N. crassa* mutant having a single amino acid change in the β-1,3-glucan synthase was isolated and the gene named *do* (*doily*) (McCluskey et al., 2011). The doily mutant has a tight colonial morphology. The *N. crassa* glucan synthase, FKS-1, is encoded by *ncu06871* and the glucan synthase has fourteen putative multiple transmembrane domains and a glucan synthesis domain that attaches a single glucose residue to the non-reducing end of a β-1,3-glucan polymer (Sanchez-Leon and Riquelme, 2015). UDP-glucose serves as the glucose donor, and the glucan is extruded through the plasma membrane into the cell wall space via a pore formed by the transmembrane domains. A β-1,3-glucan synthase regulatory subunit, COT-2 or GS-1, is also highly conserved. The *N. crassa gs-1* gene (*ncu04189*) was initially defined by mutants which lacked glucan synthase activity (Enderlin and Selitrennikoff, 1994; Tentler et al., 1997) and the *cot-2* mutation was isolated as a temperature sensitive colonial mutant (Garnjobst and Tatum, 1967). The RHO-1 GTPase (NCU01484) associates with FKS-1 and functions to regulate its activity (Richthammer et al., 2012). FKS-1 and GS-1 have been localized to the macrovesicle ring of the Spitzenkorper and to the plasma membrane at the hyphal tip (Verdin et al., 2009; Sanchez-Leon and Riquelme, 2015).

## Mixed β-1,3/β-1,4 Glucans (Lichenin)

The linkage analysis of the *N. crassa* vegetative cell wall shows that between 15 and 20% of the glucoses in the wall have a 1,4 linkage and lichenin has been shown to be present as defined by a monoclonal antibody directed against lichenin (Ao and Free, 2017; Kar et al., 2019). Lichenin is defined as a polysaccharide with a repeating β-1,4-glucose-β-1,4-glucose-β-1,3-glucose trisaccharide (Perlin and Suzuki, 1962). Lichenin was initially identified in the lichen-forming ascomycete *Cetraria islandica* (Icelandic moss) as a long linear polysaccharide. Lichenin has been shown to be located in the fungal cell wall and in the extracellular matrix formed by the ascomycete cells in the lichen (Honegger and Haisch, 2001). Based on this structure and assuming that all of the 1,4-linked glucose in the cell wall linkage analysis come from lichenin, lichenin would represent approximately 25% of the *N. crassa* vegetative cell wall mass (Kar et al., 2019). It has been shown that lichenin functions as the polysaccharide through which cell wall glycoproteins are cross-linked into the cell wall (Kar et al., 2019). Lichenin may also function to cross-link the β-1,3-glucan together into a matrix structure, but this has not been experimentally verified. In *S. cerevisiae* and *C. albicans*, β-1,6-glucan has been implicated in cross-linking both the β-1,3-glucans and the cell wall proteins into a cell wall matrix (Lu et al., 1995; Kollar et al., 1997; Kapteyn et al., 2000). It is interesting to note that *S. cerevisiae* and *C. albicans* lack lichenin and use β-1,6-glucan to cross-link glycoproteins and β-1,3-glucan into the cell wall while *N. crassa* lacks β-1,6-glucan and uses lichenin to cross-link glycoproteins into the cell wall. As a cross-linking polymer, lichenin is present throughout the middle portion of the cell wall (Figure 1).

Mixed β-1,3-/β-1,4- glucans have been found in several filamentous fungi but the proteins involved in their synthesis have not been defined. There are two plausible ways that mixed polymers could be produced. One possibility would be to have a plasma membrane localized glucan synthase, similar to the chitin and β-1,3-glucan synthases that synthesizes their polymers and extrude them through the plasma membrane. A second possibility would be to have two or three glycosyltransferases produce the polymer by the reiterative addition of glucose residues. In this scenario, the mixed β-1,3-/β-1,4- glucan could be produced in the Golgi apparatus and be secreted through the canonical secretory pathway.

Glycosyltransferase type 2 enzymes function to make polymers having β-1,4-glucose bonds (cellulose synthase type enzymes) and would therefore be considered as likely candidates for lichenin synthases. The *N. crassa* genome contains 7 genes (*cps-1/ncu00911*, *ncu09875*, *ncu08226*, *ncu04223*, *ncu03240*, *ncu09906*, and *ncu04167*) that might be considered as plausible glycosyltransferase type 2 enzymes. The information available on the expression of these genes shows that *cps-1*/*ncu00911* and *ncu03240* are highly expressed in the vegetative hyphae, and the other genes are highly upregulated during perithecium development, suggesting that they might play roles in cell wall formation during female development (Liu et al., 2017). Based on their expression in vegetative hyphae, where lichenin has been shown to be present in the cell wall, CPS-1 and NCU03240 would be considered as the most likely candidates for being lichenin synthases.

CPS-1 contains 510 amino acids and has a signal peptide, a glycosyltransferase domain and two transmembrane domains near its carboxyl terminus. Deletion of *cps-1* (*ncu00911*) gives rise to a cell wall defect that affects all aspects of the *N. crassa* life cycle (Fu et al., 2014a). The rate of vegetative growth is dramatically reduced in the mutant and the mutant is unable to produce aerial hyphae and conidia. The mutant is also unable to form perithecia. When grown in liquid medium the Δ*cps-1* mutant grows in a tight colonial form and releases large amounts of cell wall proteins into the medium (Fu et al., 2014a). Since lichenin is needed for the cross-linking of cell wall proteins into the cell wall (Ao and Free, 2017), the release of cell wall proteins into the medium suggests that *cps-1* might encode a lichenin synthase. However, sugar linkage analysis of the glucan remaining in the mutant cell wall shows the presence of 1-4 linked glucose residues, indicating that some mixed β-1,3/β-1,4-glucan is still present in the Δ*cps-1* mutant (Fu et al., 2014a).

The second likely potential lichenin synthase, NCU03240, is a 651 amino acid protein with five transmembrane domains and a centrally located glycosyltransferase domain. The *ncu03240* deletion mutant is found in the Neurospora deletion library as a heterokaryon (a cell with a mixture of wild type and mutant nuclei) and efforts to isolate the homokaryon mutant (cell containing only mutant nuclei) have not been successful. This suggests that the deletion mutant is inviable, a phenotype that would be consistent with the loss of a major cell wall component.

The current available information leaves open several possibilities, including: (1) that *cps-1/ncu00911* and *ncu03240* encode two lichenin synthases and they have overlapping, partially redundant activities, (2) that CPS-1/NCU00911 and NCU03240 synthesize two different glucans, one of which might be lichenin, and (3) that neither *cps-1* nor *ncu03240* encode a lichenin synthase, but encode other cell wall polysaccharide synthases. Although the data doesn’t definitely identify either CPS-1 or NCU03240 as being a lichenin synthase, it clearly demonstrates that both of these glycosyltransferases plays critical roles in the synthesis of the *N. crassa* vegetative hyphal cell wall. Clearly, there is still much to be learned about the synthesis of mixed β-1,3-/β-1,4-glucans and the roles they play in *N. crassa* cell wall formation.

## α-1,3-Glucans

α-1,3-glucans have been identified in a variety of fungal cell walls, including *S. pombe*, *C. neoformans*, *A. fumigatus*, and *N. crassa* (Hochstenbach et al., 1998; Beauvais et al., 2005; Grun et al., 2005; Maubon et al., 2006; Reese et al., 2007; Fontaine et al., 2010; Fu et al., 2014b). The α-1,3-glucan has been shown to be localized in the outer layers of the *Histoplasma capsulatum* yeast cell wall, where it functions to shield the underlying β-1,3-glucan from the host immune system (Rappleye et al., 2007). In the *Aspergillus nidulans* cell wall the α-1,3-glucan is in the outer layer of the cell wall, where it facilitates hyphal cell aggregation (Miyazawa et al., 2018). In addition to being produced in these fungi, α-1,3-glucan synthase genes are found in a number of additional fungal genomes suggesting that the glucan is made by a wide variety of fungi. In some cases, multiple α-1,3-glucan synthase paralogs are encoded in the genome. The α-1,3-glucan synthases have multiple transmembrane domains and a synthase domain located on the cytosolic face of the plasma membrane. Like the β-1,3-glucan synthases, the synthase domain is thought to utilize UDP-glucose as a substrate and attaches a glucose residue to the non-reducing end of an elongating α-1,3-glucan polymer. The elongating α-1,3-glucan is thought to be extruded though a pore formed by the transmembrane domains. No information is available about the three-dimensional structure of the α-1,3-glucan.

The *N. crassa* genome contains two α-1,3-glucan synthase genes, *ags-1* (*ncu08132*) and *ags-2* (*ncu02478*). The *ags-1* gene is responsible for the production of the α-1,3-glucan and is expressed in the aerial hyphae and conidia. AGS-1 is a large protein containing 2374 amino acids. In addition to a glucan synthase domain located on the cytosolic side of the plasma membrane, AGS-1 contains multiple transmembrane domains and an N-terminal putative glucanosyltransferase domain that might attach the α-glucan to the cell wall matrix. Mutants lacking AGS-1 have been extensively characterized (Fu et al., 2014b). The *ags-1* promoter has been used to drive expression of RFP and shown to be direct gene expression in developing aerial hyphae and conidia (Fu et al., 2014b). Antibodies directed against α-1,3-glucan demonstrate that the polymer is located in the cell wall and accessible to the antibody. The production of conidia in the *ags-1* deletion mutants was shown to be reduced by 95% and the conidia that were produced were shown to have a reduced level of viability and to be sensitive to a heat and freezing (Fu et al., 2014). Clearly the synthesis of the cell type-specific α-1,3-glucan is important for the development and viability of the conidia. The results further demonstrate that the glucan portion of the cell wall can vary dramatically during the *N. crassa* life cycle. No role has been defined for the *ags-2* gene, which is more highly expressed during perithecium development (Liu et al., 2017).

## Melanin

Many fungi produce melanin as one of their cell wall components. Melanin is a large amorphous polymer of phenolic compounds and is generated by a free-radical reaction in which the phenolics are randomly cross-linked together. Cell wall melanin plays a number of very important roles. It provides protection from UV light, desiccation, freezing, and digestion from cell wall digestive enzymes produced by other microbes (Rehnstrom and Free, 1996; Eisenman and Casadevall, 2012; Nosanchuk et al., 2015). Melanized fungal cells have been shown to be capable of survival in the soil for decades (Davis and DeSerres, 1970). Most pathogenic fungi have melanized cell walls, and the melanin has been shown to be an important virulence factor (Chumley and Valent, 1990; Langfelder et al., 2003; Talbot, 2003; Pihet et al., 2009; Eisenman and Casadevall, 2012).

There are two pathways that can function for the synthesis of fungal melanins, the dihydroxynaphthalene (DHN) pathway and the dihydroxyphenylalanine (DOPA) pathway, and *N. crassa* encodes the proteins for both pathways. The DOPA pathway seems to function for the melanization of the vegetative cell wall under stress conditions. The DOPA pathway requires a single copper-containing enzyme, tyrosinase, which converts tyrosine to dihydroxyphenylalanine (DOPA), which is unstable and spontaneously forms melanin granules. *N. crassa* tyrosinase has been purified, and its activity as a copper-containing enzyme characterized (Lerch, 1982; Kupper et al., 1989). The enzyme requires a proteolytic activation step to become enzymatically active. *N. crassa* vegetative hyphae that are exposed to stress agents produce tyrosinase and become melanized. *N. crassa* tyrosinase mutants have been isolated and characterized (Fuentes et al., 1994). Interestingly, these mutants are unable to form perithecia, the female mating structures. When used as a male parent in a mating, the tyrosinaseless mutant progeny have melanized cell walls, which demonstrates that the DOPA pathway is not used for ascospore melanization. Currently we have no explanation for why tyrosinase would be required for perithecium formation.

In *N. crassa*, the DHN pathway functions in the formation of melanin for the ascospore and for the peridium cell walls. The DHN melanin pathway has been well-characterized in *A. fumigatus* (Langfelder et al., 1998, 2003; Eisenman and Casadevall, 2012). The pathway for DHN synthesis was worked out and includes a polyketide synthase that uses acetyl-CoA and malonyl-CoA as substrates and makes a large heptameric polyketide (Langfelder et al., 1998; Tsai et al., 2001). The heptameric polyketide is then acted on by a hydrolase to generate pentameric tetrahydroxylnapthalene (THN) (Tsai et al., 2001). A THN reductase and a scytalone hydratase act on the THN to remove two of the hydroxyl groups and produce dihydroxynapthalene (DHN). A laccase then acts on the DHN to generate a free-radical form of DHN, which spontaneously reacts with other DHN molecules in a chain reaction manner to create large, amorphous melanin granules (Sugareva et al., 2006). In *A. fumigatus*, Upadhyay et al. (2016a, b) showed that all of the enzymes involved in the synthesis of the DHN are found associated with intracellular vesicles.

All of the enzymes involved in the synthesis of DHN are found encoded in the *N. crassa* genome. Mutants affected in the ability to produce DHN are unable to melanize their ascospores and perithecia, demonstrating that the pathway is responsible for melanizing these structures (Howe and Benson, 1974; Howe, 1976; Johnson, 1977; McCluskey et al., 2011; Ao et al., 2019). The genome has two paralogs for the heptaketide hydrolase and the THN reductase steps in the pathway, and a single gene for the other steps. The expression of the heptaketide hydrolases and the THN reductases occur in a tissue-type specific manner such that a single hydrolase and reductase are expressed in the ascospores, while both paralogs are expressed in the peridium (Ao et al., 2019). Experiments using enzymes tagged with GFP and RFP markers demonstrated that all of the DHN biosynthetic enzymes are found associated with intracellular vesicles (Ao et al., 2019). The laccase needed for the final step in the process was also identified. Experiments tagging the laccase with GFP and RFP markers demonstrated that the laccase has been secreted and localized to the cell wall space at the point in time when the peridium becomes melanized (Ao et al., 2019). It was concluded that melanin formation in *N. crassa* occurs “*in situ*” within the cell wall space and that the forming melanin granules encase the other cell wall components within the forming melanin.

## Glycoproteins

Glycoproteins are found as a major component in all fungal cell walls. Some of these glycoproteins are covalently attached to the cell wall matrix and are considered as integral cell wall components, while other cell wall proteins are incorporated into the wall via non-covalent bonds and can be released from the wall by SDS treatment. Glycoproteins that are released by SDS treatment are considered as cell wall-associated glycoproteins.

Cell wall-associated glycoproteins as well as integral cell wall glycoproteins can function in a wide variety of functions (De Groot et al., 2005; Latge, 2007; Chaffin, 2008; Klis et al., 2010; Free, 2013). Some of the integral cell wall proteins function in the cross-linking reactions described below to generate a three dimensional chitin/glucan/glycoprotein matrix. Other cell wall glycoproteins have been shown to function as adherins and help anchor the fungal cell to the substratum. Cell wall glycoproteins function as receptors for signal transduction pathways that allow the fungus to assess environmental conditions. Many cell wall glycoproteins have hydrolase activities. Some of these hydrolases may function in the remodeling of the cell wall structure to allow for modification of the cell wall and for the formation of new hyphal branches. Other cell wall hydrolases may function in nutrient acquisition by releasing sugars, amino acids, or lipids from their substrates. Cell wall glycoproteins may also play roles in protecting the fungus from other microbes. In the case of plant and animal pathogenic fungi, cell wall glycoproteins can play important roles in the infection of the host and be considered as virulence factors. Major cell wall proteins lacking enzymatic or other known functions have been ascribed a structural role, but some of these “structural proteins” may have functions that remain to be elucidated. Conversely, all of the integral cell wall proteins might be considered to have a “structural role” in that they become part of the cell wall matrix.

Proteins identified in proteomic analyses of purified cell walls have been divided into two groups, referred to as “classical” and “non-classical” cell wall proteins. Classical cell wall glycoproteins have a typical N-terminal signal sequence and are translated by ribosomes associated with the endoplasmic reticulum (ER). These proteins travel through the canonical secretory pathway, and typically have both N-linked and O-linked oligosaccharides attached to them. Proteins identified in cell wall preparations which lack a signal peptide are referred to as “non-classical” cell wall proteins. Most of these “non-classical” cell wall proteins have well-defined cytosolic functions. For example, chaperone proteins and some proteins that function in glycolysis are often found among the “non-classical” cell wall proteins that are identified in cell wall proteomic analyses. The question of whether these proteins are normal components of the cell wall or are contaminants in purified cell wall preparations remains controversial. The questions of how such proteins might be released into the cell wall space, what functions they might perform in the cell wall space, and how they might be incorporated into the cell wall haven’t been elucidated. For the purposes of this review, these “non-classical” cell wall proteins will not be further considered.

In *N. crassa* cell walls, 41 “classical cell wall proteins” have been identified by proteomic analyses. The glycoproteins present in other fungal cell walls were identified by proteomic analyses after the cell wall proteins are released from the wall by alkaline treatment, released into the medium by regenerating spheroplasts, or by having peptides released from purified cell walls by trypsin digestion. In *N. crassa*, the cell wall proteins were identified by treating purified cell wall samples with trifluoromethanesulfonic acid, which hydrolyses the glycosidic linkages in the cell wall glucans and chitin and releases free deglycosylated cell wall proteins (Bowman et al., 2006; Maddi et al., 2009). One advantage of this approach are that because of the removal of the N-linked and O-linked glycosylation, tryptic fragments that would otherwise not be able to be identified because they are glycosylated become available for identification. For highly glycosylated cell wall glycoproteins, a large fraction of the tryptic peptides are glycosylated. A second advantage of the approach is that the *N*-acetylglucosamine that is attached to the asparagine in N-linked oligosaccharides is retained, and by including asparagine-*N*-acetylglucosamine as a possible “amino acid” in the proteomic analysis, the sites of N-linked oligosaccharide addition are easily identified (Maddi et al., 2009). The identified *N. crassa* cell wall proteins are typical of those found in other fungi. They include a number of “cell wall cross-linking” enzymes, a variety of glycosylhydrolases that could be involved in cell wall remodeling or in nutrient acquisition, and a number of cell wall “structural” proteins.

Classical cell wall proteins contain a signal peptide at their N-terminus and are translocated into the ER lumen during their synthesis. As the growing polypeptides are translocated into the lumen of the ER, N-linked oligosaccharides are added. As in other eukaryotic organisms, the N-linked oligosaccharides play an important role in the assessment of protein folding and quality control. The N-linked oligosaccharide is synthesized as a 2 *N*-acetylglucosame:9 Mannose:3 Glucose structure that is attached to a dolichol phosphate moiety. The entire oligosaccharide is transferred “*en bloc*” to asparagine residues in the context of asparagine–*X*-serine or asparagine-*X*-threonine, where X can be any amino acid except proline. The glucoses on the transferred N-linked oligosaccharide function in the assessment of protein folding status and in mediating the unfolded protein response. These glucoses are trimmed in correctly folded glycoproteins.

The major elements of the glycoprotein synthesis in *N. crassa* follow those outlined above. All of the enzymes involved in the synthesis of the N-linked oligosaccharide are encoded in the *N. crassa* genome (Galagan et al., 2003; Colot et al., 2006; Deshpande et al., 2008). Deletion mutants for the several of the steps in N-linked oligosaccharide formation and transfer to nascent polypeptides are available in the knockout library as heterokaryons (isolates having wild type nuclei as well as knock out mutant nuclei in a common cytoplasm) which suggests the knockout mutations are lethal under normal growth conditions in homokaryons (cells having only knockout mutant nuclei). Classical mutations in two of the subunits of the oligosaccharide transferase have been isolated as “tiny” mutants with slow-growing, tight colonial phenotypes (McCluskey et al., 2011). This demonstrates the important roles that N-linked oligosaccharides play in the process of protein folding, protein stability, and in the incorporation of glycoproteins into the cell wall. Glycan profiling and glycan linkage analysis of the N-linked glycans present on cell wall glycoproteins in the Δ*och-1* mutant (which lacks the N-linked oligosaccharide-associated galactomannan described below) demonstrates that *N. crassa* glycoproteins have a typical 2 *N*-acetylglucosamine:9 mannose N-linked oligosaccharide (Deshpande et al., 2008; Kar et al., 2019). Trimming of some of the terminal mannoses occurs on most of the N-linked oligosaccharides and contributes to the heterogeneity seen in N-linked oligosaccharides (Kar et al., 2019).

Approximately half of the integral cell wall proteins have an attached glycosylphosphatidylinositol (GPI) anchor attached to their carboxyl terminus. GPI anchored proteins contain a typical signal peptide at their N-terminus and also contain a well-defined amino acid signal sequence at their carboxyl terminus that acts as a signal for the addition of the GPI anchor. The signal for GPI-anchor addition (the “GPI signal”) consists of a carboxyl-terminal hydrophobic domain separated by a short stretch of hydrophilic amino acids from an attachment site termed the omega site, where the protein is cleaved and the GPI anchor is added (Ferguson, 1999; Eisenhaber et al., 2003). The GPI anchor is added in the ER immediately after protein synthesis is completed. The GPI anchor plays an important role in trafficking these proteins to the cell wall. The GPI anchor contains two or three attached lipids and functions to tether or anchor the protein in the lumen leaflet of the secretory pathway organelles and to the outer leaflet of the plasma membrane. In the fungi, virtually all GPI anchored proteins are integral cell wall glycoproteins.

The pathway for the synthesis of the GPI anchor was originally elucidated using *S. cerevisiae* temperature-sensitive mutants, Trypanosomes, and mutant cultured vertebrate cells (Ferguson, 1999; Eisenhaber et al., 2003). The *N. crassa* pathway has also been examined (Bowman et al., 2006, 2009). Mutations affecting most of the steps in the *N. crassa* GPI anchor biosynthetic pathway have been characterized and the major GPI-anchored cell wall proteins have been identified and characterized (Bowman et al., 2006). Deletions for the steps in GPI anchor biosynthesis are lethal in *S. cerevisiae*, and the pathway was characterized using temperature-sensitive mutants, but the equivalent *N. crassa* deletion mutants are viable and grow with an extremely tight colonial morphology.

As the protein passes through the secretory pathway, further post-translational modifications occur. O-linked oligosaccharides are added to multiple serine and threonine sites in the glycoproteins. In fungi, these O-linked oligosaccharides usually have a mannose attached to the serine or threonine and contain additional mannose and/or galactose residues. O-linked glycosylation is important for the stability and folding of fungal glycoproteins (Shental-Bechor and Levy, 2008; Prates et al., 2018). *S. cerevisiae* contains a number of well-characterized protein:mannosyl transferases (PMT enzymes) that add the initial mannose residue to serine and threonine sites. These various PMT enzymes have differing specificities for their glycoprotein substrates (Girrbach and Strahl, 2003). Additional mannose and/or galactose residues are added by mannosyltransferases and/or galactosyltransferases in the ER and Golgi apparatus. In *S. cerevisiae*, the Mnt-1p mannosyltransferase adds the second mannose to the O-linked oligosaccharides (Hausler et al., 1992). These same steps in generating O-linked oligosaccharides occur in *N. crassa*. The *N. crassa* genome contains 3 genes encoding PMT enzymes (*ncu01912*, *ncu01648*, and *ncu09332*). Knockout mutations for *ncu01912* and *ncu09332*, are found in the deletion library as heterokaryons and there is no deletion mutant available for *ncu01648*. This strongly suggests that the addition of O-linked oligosaccharides is important for glycoprotein function and stability. *N. crassa mnt-1* mutants have been isolated and characterized (Bowman et al., 2005). The mutants grow with a tight colonial morphology and are unable produce conidia and perithecia, demonstrating the importance of O-linked glycosylation (Bowman et al., 2005). The severe growth phenotype of the *mnt-1* mutants is best understood from the viewpoint that the formation of the O-linked oligosaccharide is affected on all of the cell wall and secreted proteins. As a result, many of the cell wall proteins are being degraded. The *mnt-1* mutant cell wall is therefore deficient in several cell wall glycoproteins and is severely compromised.

**FIGURE 2:**
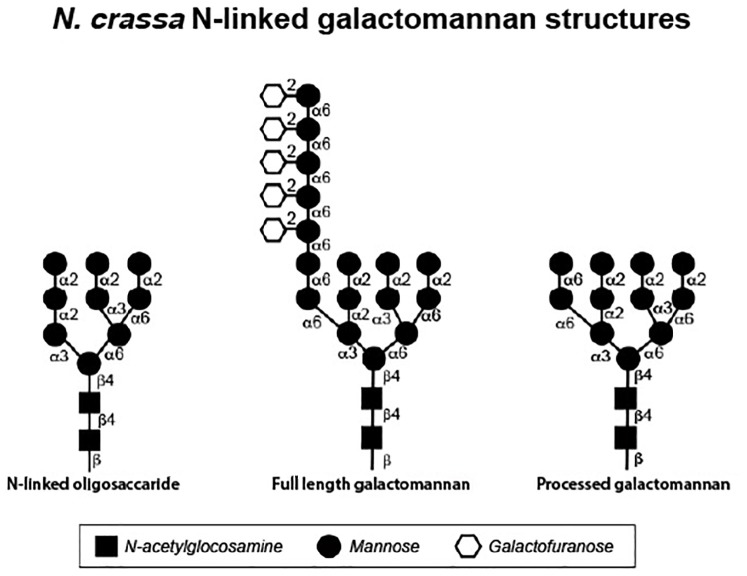
Structures of *N. crassa* N-linked oligosaccharides as determined by glycan profiling experiments. The N-linked oligosaccharide **(left)** was isolated from cell wall glycoproteins synthesized by the Δ*och-1* mutant, which is unable to elaborate the galactomannan. The full length galacatomannan **(middle)** was isolated from cell wall glycoproteins synthesized by the Δ*dfg-5*,Δ*dcw-1* mutant, which lacks the α-1,6-mannanases needed to process the galactomannan. The processed galactomannan **(right)** was isolated from cell wall glycoproteins synthesized by the Δ*gel-1*,Δ*gel-2*,Δ*gel-5* mutant, which is unable to incorporate the processed galactomannan into the cell wall.

Yet another important post-translational modification found on fungal cell wall glycoproteins is the generation of a galactomannan structure (in filamentous fungi) or an outer chain mannan structure (in *S. cerevisiae* and *C. albicans*) associated with the N-linked oligosaccharide. The synthesis of these oligosaccharide structures begins with the addition of a mannose to a particular site on the N-linked oligosaccharide by the OCH-1 mannosyltransferase (Nakayama et al., 1992) (Figure 2). Additional mannoses are then added by a complex of enzymes to create an α-1,6-mannose chain (Hall and Gow, 2013). In the creation of the yeast outer chain mannan, the α-1,6-mannose chain can be well over 100 residues in length, while the a-1,6-mannose chain is much shorter for the *N. crassa* galactomannan (Ao and Free, 2017; Kar et al., 2019). Side chains are then added to the α-1,6-mannose backbone to create the outer chain mannans or galactomannans. Multiple variations of the side chains have been seen in outer chain mannans. In *S. cerevisiae* and *C. albicans*, many of the side chains have an α-1,2-mannose-α-1,3-mannose structure but other side chains have been identified (Hall and Gow, 2013). In the filamentous fungi, galactofuranose residues are found in the side chains of the galactomannan. The *N. crassa* galactomannan structure has been characterized for galactomannans released from the cell wall and for galactomannans released from cell wall glycoproteins in glycan profiling experiments (Leal et al., 1996; Kar et al., 2019). The structure of the *N. crassa* full length galactomannan is shown in Figure 2. It consists of a short chain of 1,6-linked mannose residues with a single galactofuranose side chain that is attached to the mannose residues at their C2 position The galactofuranosyltransferase responsible for the addition of the galactofuranose side chain to the mannose backbone has not been identified.

In *N. crassa*, mutants affected in the formation of the galactomannan have been identified. The Δ*och-1* mutant (*ncu00609*) has a severe tight colonial morphology and has been carefully characterized (Maddi and Free, 2010). The mutant is unable to produce conidia and perithecia. During growth in a liquid medium, the Δ*och-1* mutant releases large amounts of cell wall proteins into the growth medium and analysis of the cell wall shows that the wall is deficient in cell wall proteins (Maddi and Free, 2010). This demonstrates that the galactomannan is required for the incorporation of cell wall proteins into the wall and suggests that the cell wall proteins are attached to the wall through the galactomannan. Glucan profiling of the N-linked oligosaccharides present on glycoproteins from the Δ*och-1* mutant, shows that the largest N-linked oligosaccharide present on the glycoproteins has a 2 *N*-acetylglucosamine: mannose 9 structure (Figure 2). Most of the N-linked oligosaccharides present on N-linked oligosaccharides from the Δ*och-1* mutant contain between 4 and 6 mannose residues, which indicates that mannoses are being removed from the N-linked oligosaccharides after they are transferred onto target glycoproteins.

## Biogenesis of the Cell Wall as a Three-Dimensional Matrix

Glucanosyltransferases carry out the key reactions needed to cross-link the cell wall glucans and chitins together. The genes encoding these enzymes are found as multigene families and are restricted to fungal genomes. Multiple members of these multigene families are expressed in a single cell type, which creates a situation of redundancy in their cross-linking activities. This redundancy is thought to help insure that a well-formed cell wall is generated across the spectrum of environmental conditions in which the fungus can grow, with different family members being optimally active in different environments (Fonzi, 1999; Calderon et al., 2010). Different combinations of these genes are also expressed in the various cell types generated during the fungal life cycle. The glucanosyltransferases carry out two closely related reactions. They first function as a glucan hydrolase to cleave a cell wall glucan near the reducing end of the glucan. These enzymes contain a characteristic arrangement of glutamate or aspartate residues that participate in the cleavage reaction. During the reaction, the newly generated reducing end of the cleaved glucan becomes covalently attached to a glutamate or aspartate in the active site. The reaction releases a small oligosaccharide from the reducing end of the glucan (Mouyna et al., 1998). In a second reaction, the enzymes function as glucanosyltransferases. The enzymes bind a second glucan and transfer the cleaved glucan from their active site to the second glucan. These transferase reactions can occur in such a way as to transfer the cleaved glucan onto the middle of a second polymer to create a cross-linked matrix with the branch points in the matrix having been created by transferase reactions. These glucanosyltransferases have specificity for both the donor glucan and the acceptor glucan. For example, members of the GH16 family of glycosylhydrolases/glycosyltransferases have been shown to function in cross-linking β-1,3-glucan and β-1,6-glucan (donors) to chitin (receptor) polymers (Pardini et al., 2006; Cabib et al., 2007; Hartl et al., 2011). In *S. cerevisiae*, members of the GH17 family of glucosylhydrolases/glycosyltransferases have been shown to have specificity for cross-linking β-1,3-glucan to β-1,3-glucans (Goldman et al., 1995; Gastebois et al., 2010b). In *S. cerevisiae*, *C. albicans*, and *A. fumigatus*, members of the GH72 family of glucanosyltransferases have also been demonstrated to be able to cross-link β-1,3-glucans together (Hartland et al., 1996; Hurtado-Guerrero et al., 2009; Mazan et al., 2011). A three-dimensional cell wall matrix of chitin and glucan is generated as these different glucanosyltransferases cross-link the cell wall chitin and glucan molecules together. The GH16, GH17, and GH72 families of glycosylhydrolases are common to virtually all fungal cells walls, and have been shown to be important for cross-linking cell wall components. Members of the GH76 family of α-1,6-mannanases are also found in all fungal cell walls. We will discuss each of these families of enzymes and how each of them is thought to function in the formation of *N. crassa* cell walls. Our research focus has been on how the cell wall glycoproteins are incorporated into the cell wall and the information available about the *N. crassa* cross-linking enzymes reflects this bias.

## The Gh16 Family of Glucanosylhydrolases/Glucanoysltransferases

In *S. cerevisiae* and *C. albicans*, mutational analysis shows that GH16 enzymes function in cross-linking β-1,6-glucan to the cell wall chitin (Pardini et al., 2006; Cabib et al., 2007). In *S. cerevisiae*, there are three GH16 enzymes, Crh1p, Crh2p, and Crr1p. Deletion of Crh1p and Crh2p is needed to create a cell wall defect, indicating that the two enzymes function in a redundant manner to attach β-1,6-glucan to the cell wall chitin. Similarly, *C. albicans* contains three GH16 enzymes, Crh11p, Crh12p, and Utr2p. The proteins function in a redundant manner and deletion of all three genes generates a cell wall defect (Pardini et al., 2006). A GH16 enzyme, Eng2p, has been characterized in *A. fumigatus* and shown to have β-1,3-glucanase and β-1,3-glucanosyltransferase activities (Hartl et al., 2011). These results demonstrate the importance of the GH16 family of enzymes for the formation of a functional cell wall in yeast, and that the yeast GH16 enzymes function in a redundant manner to cross-link β-1,6-glucans to the cell wall chitin polymers. The results also suggest that there are substrate specificity differences between the *A. fumigatus* enzyme and the yeast enzymes.

The *N. crassa* genome encodes 15 GH16 family glucanosyltransferases (NCU01353, NCU04168, NCU04431, NCU4959, NCU5686, NCU05789, NCU05974, NCU06504, NCU07134, NCU08072, NCU09117, NCU09904, NCU00061, NCU00233, and NCU09672). Different combinations of these GH16 glucanosyltransferases are expressed in the different cell types found in the *N. crassa* life cycle (Lehr et al., 2014; Wang et al., 2014; Liu et al., 2017). Deletion mutants for the GH16 genes are available in the deletion library and all of these deletion mutants have a wild type growth morphology.

## The Gh17 Family of Glucanosylhydrolases/Glucanosyltransferases

The GH17 family of enzymes have been extensively studied in *S. cerevisiae*, *C. albicans*, and *A. fumigatus*. The enzymatic activity of purified Bgl2p, a GH17 enzyme from *S. cerevisiae*, has been characterized (Klebl and Tanner, 1989; Goldman et al., 1995; Gastebois et al., 2010b). In *in vitro* reactions, the enzyme was shown to be able to cleave a disaccharide from the reducing end of a β-1,3-glucan and to transfer the glucan to the 6 position at the non-reducing end of a second β-1,3-glucan to generate a “kinked” polymer. Two GH17 enzymes, AfBgt1p and AfBgt2p, have been characterized from *A. fumigatus* (Gastebois et al., 2009, 2010a,b). AfBgt1p, like the *S. cerevisiae* Bgl2p, was able to generate a “kinked” glucan by transferring a β-1,3-glucan to the 6 position at the non-reducing terminus of a second β-1,3-glucan in an *in vitro* reaction. AfBgt2p had a slightly different activity. In the *in vitro* assay, the enzyme was able to transfer a β-1,3-glucan to the 6 position on a glucose residue in the middle of an acceptor β-1,3-glucan to generate a branched glucan molecule (Gastebois et al., 2010a, b). The *A. fumigatus* Af*BGT1*, Af*BGT2* double mutant does not have an obvious cell wall defect, which suggests there are other enzymes that also act in cross-linking the β-1,3-glucans together and that the wall has a redundancy of β-1,3-glucan cross-linking enzymes (Gastebois et al., 2009). In *A. fumigatus*, the GH72 family is an obvious possibility for additional β-1,3-glucan cross-linking activity.

The *N. crassa* genome encodes 3 members of the GH17 family of β-1,3-glucan cross-linking enzymes, BGT-1 (NCU06381), BGT-2 (NCU09175), and BGT-3 (NCU09326). Deletion mutants for all three genes are available in the deletion library and these mutants have a wild type growth morphology. BGT-1 and BGT-2 are GPI-anchored proteins and their location on the cell wall and in secretory vesicles in vegetative hyphae and in conidia has been characterized by Martinez-Nunez and Riquelme (2015). Liu et al. (2017) found that BGT-1 and BGT-2 are expressed at high levels in the developing ascospores, while BGT-3, which does not have a GPI anchor, is expressed at high levels during vegetative growth. Martinez-Nunez and Riquelme (2015) demonstrated that the Δ*bgt-1*, Δ*bgt-2* double mutant had a normal morphology, but showed an increased resistance to calcofluor white and congo red, suggesting that the cell wall was affected in the double mutant. The data leaves open the possibility that the three *N. crassa* GH17 glucanosyltransferases are redundant and a triple mutant is needed to demonstrate the role the enzymes play in cell wall formation. Another possibility is that the *N. crassa* GH17 enzymes, like the *A. fumigatus* GH17 enzymes, are not vital for the formation of the cell wall. Further experiments are needed to define the role of the GH17 family enzymes for the formation of the *N. crassa* cell wall.

## The Gh76 Family of α-1,6-Mannanases

The GH76 α-1,6-mannanases are found in virtually all fungal cell walls. Two GH76 enzymes, Dfg5p and Dcw1p were shown to be important for the formation of the *S. cerevisiae* cell wall and the double mutant is inviable (Mosch and Fink, 1997; Kitagaki et al., 2002; Kitagaki et al., 2004). A similar situation exists in the diploid fungus, *C. albicans*, where the homozygous loss of both CaDfg5p and CaDcw1p alleles is a lethal event (Spreghini et al., 2003). Clearly the GH76 family of α-1,6-mannanases play a vital role in the formation of the cell wall.

The *N. crassa* genome encodes 9 GH-76 α-1,6-mannanase family members. As with the other families of cell wall cross-linking enzymes, different combinations of the GH76 family genes are expressed in the various cell types that define the *N. crassa* life cycle. Two of the GH76 α-1,6-mannanases, DFG-5 (NCU03770) and DCW-1 (NCU08127), are needed for the formation of the cell wall of the vegetative hyphae (Maddi et al., 2012).

The *N. crassa* GH76 enzymes, DFG-5 and DCW-1 have been characterized (Maddi et al., 2012). Unlike the other enzymes discussed in the section, the data strongly suggests that the GH76 enzymes do not function as mannan transferases, but rather function solely as α-1,6-mannan hydrolases. The Δ*dfg-5* mutant has a restricted, semi-colonial pattern of vegetative growth and the Δ*dcw-1* mutant has a subtle defect in vegetative hyphal morphology. The Δ*dfg-5*Δ*dcw-1* double mutant has a tight colonial morphology, demonstrating that the two enzymes have redundant, partially overlapping activities. The double mutant has been shown to release large amounts of cell wall proteins into the growth medium and to have a cell wall that is deficient in glycoproteins (Maddi et al., 2012). This strongly suggests that DFG-5 and DCW-1 function in the incorporation of cell wall proteins into the cell wall. Glycan profiling and sugar linkage analyses of the N-linked glycan found on the glycoproteins of mutant isolates provides evidence that DFG-5 and DCW-1 function as α-1,6-mannanases to cleave the α-1,6-mannose backbone of the N-linked oligosaccharide-associated galactomannans (Kar et al., 2019). The deduced N-linked oligosaccharide-galactomannan structure from the glycoproteins from the Δ*dfg-5*Δ*dcw-1* mutant is shown in Figure 2. It represents a “full length” galactomannan and has an α-1,6-mannan backbone containing approximately 7 mannose residues. The deduced structure for the N-linked glycans that have been processed by DFG-5 and DCW-1 is also shown in Figure 2. It is much smaller than the “full length” galactomannan, and is only 2 sugars larger than the N-linked oligosaccharide lacking the galactomannan. The results indicated that DFG-5 and DCW-1 function in processing the galactomannan and are needed for the incorporation of cell wall proteins into the cell wall.

## The Gh72 Family of Glucanosyltransferases

Members of the GH72 family of glucanosyltransferases have been extensively studied in *S. cerevisiae*, *C. albicans*, *A. fumigatus*, *S. pombe*, and *N. crassa* (Hartland et al., 1996; Ram et al., 1998; Fonzi, 1999; Mouyna et al., 2000b, 2005; Caracuel et al., 2005; Ragni et al., 2007a, b; Gastebois et al., 2009, 2010a,b; Hurtado-Guerrero et al., 2009; de Medina-Redondo et al., 2010; Mazan et al., 2011; Sillo et al., 2013; Popolo et al., 2017; Samalova et al., 2017). The fungal GH72 glucanosyltransferases can be subdivided into two groups, those with a carboxyl terminal carbohydrate-binding domain and those that lack such a domain (Ragni et al., 2007b). GH72 enzymes from *S. cerevisiae* (Gas1p, Gas2p, Gas4p, and Gas5p), *C. albicans* (Phr1p and Phr2p), *A. fumigatus* (Gel1p, Gel2p, and Gel4p), and *S. pombe* (Gas1p, Gas2p, Gas4p, and Gas5p) have all been produced by recombinant DNA technology, purified, and characterized (Mouyna et al., 2000a, b; Carotti et al., 2004; Ragni et al., 2007b; Hurtado-Guerrero et al., 2009; de Medina-Redondo et al., 2010; Mazan et al., 2011; Kovacova et al., 2015; Raich et al., 2016). These recombinant glucanosyltransferases have been shown to be able to cleave a β-1,3-glucan and to transferase the β-1,3-glucan to the non-reducing end of a second β-1,3-glucan. The reaction can lengthen and shorten β-1,3-glucans and has been proposed to function in generating glucans of the proper lengths for incorporation into the cell wall. The enzyme-β-1,3-glucan intermediate has been observed for Gas2p (Hurtado-Guerrero et al., 2009; Raich et al., 2016). Recent evidence suggests that the *S. cerevisiae* Gas1p and *A. fumigatus* Gel4p enzymes are capable of transferring β-1,3-glucan to the 6 position of a glucose residue in the middle of a second β-1,3-glucan to create a branched structure appropriate for an interconnected β-1,3-glucan matrix (Aimanianda et al., 2017).

The x-ray crystal structure of the purified recombinant *S. cerevisiae* Gas2p glucanosyltransferase with an associated β-1,3-glucan has been elucidated and is helpful in evaluating how the enzyme might work (Hurtado-Guerrero et al., 2009; Raich et al., 2016). The crystal structure contains a long cleft into which the β-1,3-glucan fits and makes contacts with several amino acids. The active site is defined by a pair of glutamate residues (E176 and E275), which function in cleaving the glucan and producing an enzyme:glucan covalent intermediate. The glutamate residue participates in the formation of the covalent bond. Based on the data from the *S. cerevisiae*, *C. albicans*, and *A. fumigatus* systems, it is clear that the GH72 glucanosyltransferases can cleave β-1,3-glucan and participate in its transferase to a second polysaccharide. The data has been interpreted as indicating that the GH72 glucanosyltransferases function to cross-link β-1,3-glucans together.

The studies on the *N. crassa* GH72 family of glucanosyltransferases suggests that these glucanosyltransferases may have a second, related enzymatic function – that of cross-linking cell wall proteins into the cell wall. The *N. crassa* genome encodes a family of five GH72 glucanosyltransferases (GEL-1/NCU08909, GEL-2/NCU07253, GEL-3/NCU01162, GEL-4/NCU06850, and GEL-5/NCU06781). Four of these were found to be expressed in proteomic analyses, GEL-1, GEL-2, GEL-3, and GEL-5 (Maddi et al., 2009, 2012; Maddi and Free, 2010; Ao et al., 2016). Deletion mutants for all of these are in the Neurospora deletion library, and all of the single deletion mutants have a wild type morphology. The deletion mutants were shown to be less sensitive to Trichoderma cell wall lysing suggested they had alterations in their cell wall structure (Kamei et al., 2013). All possible combinations of single, double, triple and quadruple deletion mutants have been generated and characterized (Ao and Free, 2017). Triple mutants lacking GEL-1, GEL-2 and GEL-3 grow poorly, are unable to form conidia, and have a tight colonial morphology when grown in liquid medium. The Δ*gel-1*Δ*gel-2*Δ*gel-5* triple mutant and the quadruple mutant have an even more severe phenotype and grow with a tight colonial morphology under all conditions. The Δ*gel-1*Δ*gel-2*Δ*gel-5* mutant phenotype is indistinguishable from that of the Δ*och-1* mutant and the Δ*dfg-5*Δ*dcw-1* double mutant. The Δ*gel-1*Δ*gel-2*Δ*gel-3* and Δ*gel-1*Δ*gel-2*Δ*gel-5* mutants release large amounts of cell wall proteins into the growth medium and their cell walls are deficient in cell wall proteins (Ao and Free, 2017). In a series of experiments to elucidate the function(s) of the GEL1, GEL-2 and GEL-5 glucanosyltransferases, the cell wall proteins from the Δ*och-1* mutant, the Δ*dfg-5*Δ*dcw-1* mutant, and the Δ*gel-1*Δ*gel-2*Δ*gel-5* mutant were assayed for *in vitro* glucanosyltransferase activity. Using experiments in which combinations of cell wall proteins were mixed with β-1,3-glucan or lichenin, it was determined that lichenin (but not β-1,3-glucan) was transferred to cell wall proteins when the assays contained a source of glucanosyltransferase (from the Δ*och-1* mutant or from the Δ*dfg-5*Δ*dcw-1* mutant) and source of cell wall proteins containing the “processed” galactomannan (from the Δ*gel-1*Δ*gel-2*Δ*gel-5* mutant). All three components were needed for the transfer of lichenin to the cell wall proteins (Kar et al., 2019). Cell wall proteins without a galactomannan (from the Δ*och-1* mutant) and cell wall proteins with a full-length unprocessed galactomannan (from the Δ*dfg-5*Δ*dcw-1* mutant) are not able to act as lichenin acceptors in the assay. It was concluded that GEL-1, GEL-2, and GEL-5 can function as lichenin transferases to cross-link cell wall glycoproteins and lichenin. The activity identified for the *N. crassa* GH72 family glucanosyltransferases is similar to that ascribed for the enzymes in *S. cerevisiae*, *C. albicans*, and *A. fumigatus* in that a β-glucan is cleaved and transferred to as second polysaccharide, but the specificities of both the donor and acceptor are different. The family of GH72 glucanosyltransferases may have a much broader range of substrate specificities than previously appreciated. It is interesting to note that the GH72 glucanosyltransferase genes from *Magnaporthe oryzae*, *Fusarium oxysporum*, and *Tuber melanosporum*, three filamentous fungi related to *N. crassa*, do not complement the *S. cerevisiae gas1* mutant (Caracuel et al., 2005; Sillo et al., 2013; Samalova et al., 2017). This suggests that the GH72 enzymes of these fungi may function in cross-linking glycoproteins into their cell walls.

## Summary of How *N. crassa* Generates a Three-Dimensional Cell Wall Matrix

In generating the cell wall as a three-dimensional matrix, the three major cell wall components, chitin, glucans and glycoproteins, all need to be joined together. The cross-linking of chitin, glucans, and glycoproteins is vital for the creation of a functional cell wall. Although much remains to be elucidated, it is clear that the cell wall biosynthetic enzymes we have discussed above have the capacity to generate a cross-linked chitin/glucan/glycoprotein matrix. In the *N. crassa* cell wall, the β-1,3-glucans and lichenin are the most abundant glucan component and represent approximately 65% of the total cell wall mass (Maddi and Free, 2010; Maddi et al., 2012; Fu et al., 2014a; Ao and Free, 2017). Chitin and the cell wall glycoproteins are attached to the cell wall glucans in *N. crassa* and other fungi. The GH16 family of glucanosyltransferases from *S. cerevisiae*, *C. albicans*, and *A. fumigatus* have been shown to have the capacity to cross-link glucan to chitin (Pardini et al., 2006; Cabib et al., 2007; Hartl et al., 2011), and it is presumed that they function in this capacity in *N. crassa*. Which of the major glucan polymers, β-1,3-glucan or lichenin, is used in cross-linking the *N. crassa* chitin to the matrix has not been experimentally addressed. Cross-linking of the β-1,3-glucans and lichenin together would be expected to be a critical step in the formation of the cell wall. The *S. cerevisiae* GH17 family of glucanosyltransferase Bgl2p has been shown to be able to cross-link β-1,3-glucans together and the *Neurospora* GH17 enzymes are likely to function in cross-linking the *N. crassa* cell wall together. The question of how the α-1,3-glucan found in the *N. crassa* aerial hyphae and conidia is cross-linked into the cell wall has not been experimentally examined.

The incorporation of cell wall glycoproteins into the wall has been extensively examined in *N. crassa*. As shown in Figure 2, the *N. crassa*, cell wall glycoproteins are post-translationally modified by the addition of a galactomannan to their N-linked oligosaccharides (Maddi and Free, 2010). The galactomannan is subsequently cleaved/processed by the α-1,6-mannanases DFG-5 and DCW-1 (Maddi et al., 2012; Kar et al., 2019). The processed galactomannan is then used as the acceptor polysaccharide by the GH72 family of glucanosyltransferases (lichenin transferases) (Kar et al., 2019). These enzymes cleave lichenin and attach it to the processed galactomannan, which effectively cross-links the glycoproteins into the cell wall. The method of cross-linking the glycoproteins into the wall is virtually identical to the process used to cross-link the other cell wall components together. The enzymes needed to cross-link the processed galactomannan into the wall could have easily evolved from glucan cross-linking transferases through small changes in their donor-binding and acceptor-binding clefts to accommodate a new set of donor and acceptor polysaccharides. Although the general principles defined in *N. crassa* for cross-linking glycoproteins into the cell wall may be generally applicable, there will clearly be some differences between different fungal species. For example, the DFG-5 and DCW-1 enzymes are needed for incorporation of cell wall proteins in *C. albicans* (Ao et al., 2015), but *C. albicans* lacks lichenin. A different donor glucan would be needed to attach cell wall proteins in *C. albicans*. The available evidence indicates that β-1,6-glucans are used in attaching glycoproteins into the cell wall in both *C. albicans* and *S. cerevisiae* (Lu et al., 1995; Kollar et al., 1997; Kapteyn et al., 2000). It is also important to recognize that other modes of attaching glycoprotein to the cell wall have been observed. For example, in *S. cerevisiae*, the attachment of a β-1,6-glucan to the GPI anchor present on GPI-anchored cell wall glycoproteins has been observed, which would tether GPI-anchored proteins into the cell wall structure (Kollar et al., 1997; Kapteyn et al., 2000). While some fungi may have multiple ways of attaching glycoproteins to the wall, the incorporation of glycoproteins into the *N. crassa* cell wall seems to be totally dependent upon the processed galactomannan route described above.

The incorporation of melanin into the fungal cell wall is an important process, and is vital to the survival of the melanized cells. For *N. crassa* the question of how melanin is incorporated into the cell wall has been answered by the demonstration that LACM-1, the laccase needed for the final step in the process of melanin formation is located in the cell wall space in developing perithecia at the point in time when melanin is being formed (Ao et al., 2019). At the same point in time, the enzymes involved in the synthesis of DHN, the melanin precursor are located on intracellular vesicles. The results indicate that DHN is synthesized in intracellular vesicles. The DHN is then secreted into the cell wall space, where LACM-1 acts on the DHN to generate melanin granules (Ao et al., 2019). The melanin is made “*in situ*” and as the granules form they encase the other cell wall components.

## Changing the Cell Wall Throughout the *N. crassa* Life Cycle

One of the interesting aspects of the *N. crassa* cell wall is how the cell wall structure and composition changes during the life cycle of the organism. Table 1 shows the major cell wall proteins identified via proteomic analyses of the vegetative hyphae and conidia (Bowman et al., 2006; Maddi et al., 2009, 2012; Maddi and Free, 2010; Ao et al., 2016). What is evident is that different combinations of cell wall glycoproteins are expressed in the two cell types. Seventeen of the cell wall glycoproteins in the vegetative cell wall are also present in the conidial cell wall. However, the vegetative hyphal cell wall contains seven major vegetative cell wall glycoproteins which are missing from the conidia cell wall. While sharing seventeen cell wall glycoproteins with the vegetative hyphal cell wall, the conidial cell has seventeen cell wall glycoproteins that are not found in the vegetative cell wall (Table 1). Interestingly, most of these additional conidia-specific glycoproteins lack a GPI-anchor. An analysis of the deletion mutants for these conidia-specific cell wall glycoproteins showed that two of them, CGL-1/NCU07523 and NAG-1/NCU10852 play significant roles in conidia development. CGL-1 is a β-glucanase and NAG-1 is an exochitinase, and both activities are needed to remodel the conidia cell wall between adjacent conidia to facilitate the separation of the individual conidia in a conidial chain from each other (Ao et al., 2016). The conidia-specific expression of the conidia cell wall associated hydrophobin, EAS (easily wettable)/CCG-2 (NCU08457) is yet another example of an important cell wall difference between vegetative hyphae and conidia (Bell-Pedersen et al., 1992). The EAS/CCG-2 hydrophobin forms a hydrophobic surface rodlet layer around the conidia cell wall and facilitates the dispersal of conidia in an aqueous environment. These conidia-specific cell wall proteins play important roles in the development of the conidia.

**TABLE 1:** Table of *N. crassa* cell wall proteins.

**Protein name**	**NCU#**	**GPI anchored**	**Total # of peptides**	**Cell type expression**
GH17 (β-1,3-endoglucanase)	09175	Yes	20	V and C
GH16 (β-1,3-endoglucanase transferase)	05974	Yes	23	V and C
GEL-1 (GH76 β-glucan transferase)	08909	Yes	16	V and C
GEL-2 (GH76 β-glucan transferase)	07253	Yes	14	V and C
GEL-5 (GH76 β-glucan transferase)	06781	Yes	12	V and C
CHIT-1 (endochitinase)	02184	Yes	16	V and C
ACW-1	08936	Yes	18	V and C
ACW-2	00957	Yes	9	V and C
ACW-3	05667	Yes	17	V and C
ACW-5	07776	Yes	3	V and C
ACW-6	03530	Yes	3	V and C
ACW-7	09133	Yes	7	V and C
ACW-10	03013	Yes	3	V and C
GH17 (β-1,3-endoglucanase)	09326	No	8	V and C
GH3 (β-glucosidase)	08755	No	14	V and C
CAT-3 (catalase)	00355	No	10	V and C
NCW-3	07817	No	1	V and C
GH16 (β1,3-endoglucanase)	01353	Yes	9	V
ACW-8	07277	Yes	2	V
ACW-9	06185	Yes	2	V
ACW-11	02041	Yes	1	V
ACW-12	08171	Yes	12	V
NCW-1	05137	No	18	V
NCW-2	01752	No	7	V
GEL-3 (β-glucan transferase)	01162	Yes	5	C
GH64 (β-1,3-glucanase)	01080	Yes	1	C
ACW-4	09263	Yes	1	C
ACW-13	04493	Yes	1	C
NAG-1 (β-*N*-acetyl hexosaminidase)	10852	No	10	C
CGL-1 (GH55) (β-1,3-glucanase)	07523	No	8	C
GH55 (β-1,3-glucanase)	09791	No	5	C
GH71 (α-1,3-glucanase)	06010	No	1	C
GH31 (α-glucosidase)	09281	No	1	C
NCW-4	02948	No	2	C
NCW-5	00716	No	2	C
NCW-6	00586	No	5	C
NCW-7	08907	No	2	C
NCW-8	04605	No	2	C
NCW-9	03083	No	1	C
HET-C	03125	No	4	C
RDS-1	05143	No	1	C

*The various cell wall glycoproteins that have been identified in *N. crassa* cell wall preparations. The NCU # refers to the gene number assigned in the genome sequence to the protein. The presence or absence of a GPI anchor is noted. The number of unique tryptic peptides identified is also shown. The cell type(s) in which the glycoproteins were found is given with V denoting vegetative hyphae (grown in a liquid medium) and C denoting conidia.*

**FIGURE 3:**
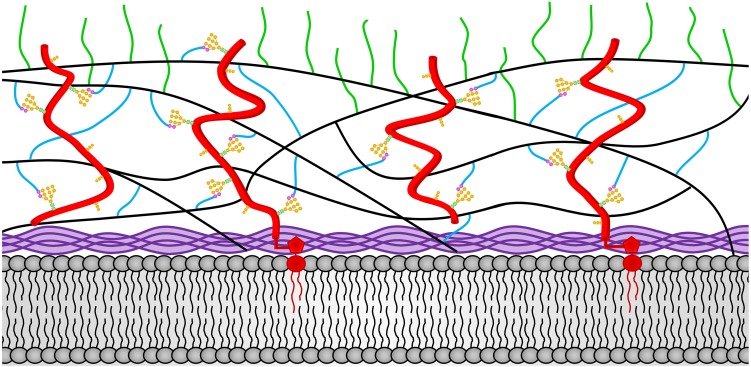
The *N. crassa* conidia cell wall. The locations of the components of the conidial cell wall are depicted. Chitin is shown in purple and is located adjacent to the plasma membrane at the bottom of the diagram. The β-1,3-glucan is shown in black and located in the middle of the cell wall. Cell wall glycoproteins are shown in red. GPI anchors are shown in red and extent into the plasma membrane. N-linked oligosaccharides are shown with *N*-acetylglucosamine residues in green squares, mannoses from the N-linked oligosaccharide shown in orange circles, and processed galactomannans shown in magenta circles. O-linked oligosaccharides are also shown in orange. Lichenin is shown in blue and is attached to the processed galactomannan and to β-1,3-glucans. The α-1,3-glucans are shown in green and are attached to β-1,3-glucan and/or lichenin at the cell wall periphery.

A second important difference between the cell wall of the vegetative hyphal cell and the conidia is found in their glucan components. The conidia contains α-1,3-glucan, which is lacking from the vegetative cell wall. Like the expression of the CGL-1 and NAG-1 cell wall remodeling enzymes, synthesis of α-1,3-glucan plays an important role in conidial development (Fu et al., 2014b). Mutants lacking α-1,3-glucan are unable to produce normal conidia. This demonstrates that the formation of conidia requires major changes in the glucan components of the cell wall as well as the expression of conidia-specific glycoproteins. Figure 3 shows a representation of the conidial cell wall with the α-1,3-glucan being localized at the periphery of the cell wall.

There are no published proteomic analyses of the cell walls produced during the sexual stages of the *N. crassa* life cycle. However, there are three RNAseq analyses of gene expression during sexual development (Lehr et al., 2014; Wang et al., 2014; Liu et al., 2017). In looking through the data from these RNAseq analyses, it is clear that members of the GH16, GH17, GH72, and GH76 gene families which are not expressed in vegetative hyphae are being expressed in the developing ascospores (sexual spores) and in the peridium (a female-derived tissue that surrounds and protects the developing ascospores). In addition to these changes in the cross-linking enzymes, many other genes encoding putative cell wall remodeling enzymes and structural proteins are being differentially expressed during the sexual stages. These changes in gene expression extend to genes encoding putative mixed β-1,3-/β-1,4- glucan synthases. Deletion mutants for the chitin synthases demonstrate that some chitin synthases are critical for the development of perithecia, ascospores, and conidia, further demonstrating that there are important cell wall differences between these different cell types (Fajardo-Somera et al., 2015). Not only are there major changes in the expression of cell wall glycoproteins and glucans, the developing ascospores and peridium cells become heavily melanized. The expression of the DHN pathway enzymes and the LACM-1 laccase are regulated in a cell-type specific manner in the developing ascospores and peridium (Ao et al., 2019). Unfortunately, deletion mutants for the different glycoproteins and glucan synthases expressed uniquely in the ascospores and peridium have not been carefully analyzed. Although the ascospore and peridium cell walls have not been characterized by proteomics, the available data makes it clear that there are major differences between the cell walls produced during sexual development and the cell walls from vegetative hyphae and conidia.

In summary, each of the different cell types in the *N. crassa* life cycle produces a cell wall with a unique combination of glycoproteins, glucans, and melanin. Some of these components, like CGL-1, NAG-1, α-1-3-glucan, and melanin, have been shown to carry out important cell-type specific functions (Fu et al., 2014b; Ao et al., 2016, 2019). There is also a cell-type expression pattern for the members of the GH16, GH17, GH72, and GH76 gene families, with different combinations of these genes being expressed in each cell type (Lehr et al., 2014; Wang et al., 2014; Ao and Free, 2017; Liu et al., 2017). Many other cell wall remodeling enzymes and structural proteins are expressed in cell-type specific fashion (Ao et al., 2016). While the glucan/chitin/glycoprotein matrix remains the basic cell wall structure throughout the entire life cycle of the fungus, this structure is being extensively modified by adding new glycoproteins, changing glucans, and/or the incorporation of melanin to control cell morphology and to facilitate cell development.

## Author Contributions

PP and SF contributed to the writing and editing of the review.

## Conflict of Interest

The authors declare that the research was conducted in the absence of any commercial or financial relationships that could be construed as a potential conflict of interest.
